# Supplementary material for: Assembly of Biologically Functional Structures by Nucleic Acid Templating: Implementation of a Strategy to Overcome Inhibition by Template Excess
Source: Molecules. 2022 Oct 12;27(20):6831. doi: 10.3390/molecules27206831 (PMC9610079; doi:10.3390/molecules27206831)
Supplement: Supplementary file 1 [file molecules-27-06831-s001.zip › molecules-1910728-supplementary.pdf]

SUPPLEMENTARY INFORMATION

Assembly of Biologically Functional Structures by Nucleic Acid Templating: Implementation of a Strategy to Overcome Inhibition by Template Excess

Matthew M. Lawler [1], James T. Kurnick [1,2] Leah Fagundes St. Pierre [1], Estelle E. Newton [1], Lenora B. Rose [1,2], and Ian S. Dunn [1 *]

[1] TriBiotica LLC, 100 Cummings Center; Suite 424-J; Beverly MA 01915; [2] Department of Pathology, Massachusetts General Hospital, Boston MA

Material included:
Supplementary Text
Tables S1-S3.
Supplementary Figures S1-S15.

Supplementary Text

Locked-NATS Theory

The participating molecules in a locked NATS reaction can be expressed as the following equilibria:

([L]cH1::H1) + T ⇌ (cH1—T::L—H1) (1)

(cH1—T::L—H1) + H2 ⇌ (cH1—T :: L—H1 :: H2) (2)

Where:

([L]cH1::H1) = First haplomer "bottle" structure, where cH1 = the internal complementary sequence to the First haplomer sequence H1; and thus cH1::H1 = the stem structure formed by hybrization of cH1 and H1, which in turn promotes the formation of the anti-target loop L, directed against (complementary to) an arbitrary target sequence T.

cH1—T:: L—H1 = target-loop duplex with destabilized stem and exposure of the First haplomer H1 sequence, recognizable by the Second haplomer H2.

Haplomer product formation is enabled by the formation of the 'unlocked' structure on the right of equation (2). The Second haplomer (H2) can only hybridize to the First haplomer (H1) after H1 has been exposed through the presence of specific template. In this circumstance, template excess not only cannot have a titration effect, but is also beneficial through shifting equilibrium (1) further to the right, thus providing more available H1 sequence.

The Tm values for the respective functional components of the Locked-NATS system must be designed to accommodate the desired system behavior, such that the Tm for (H1 :: H2) haplomer duplex < Tm (cH1 :: H1) stem duplex < Tm (T :: L) target / First haplomer Loop duplex. The Tm for the (H1 :: H2) First and Second haplomer duplex should be less than the (cH1 :: H1) stem duplex in order to minimize unwanted strand invasion events in the absence of specific target sequences, and the stem duplex in turn should be significantly less thermally stable than the (T :: L) target / First haplomer Loop duplex, to facilitate 'bottle opening' in the presence of target nucleic acid strands. Specific Tm values for the First and Second haplomer examples of Fig. 4A are: target sequence / loop (T :: L): 70˚ C; stem in First haplomer bottle (cH1 :: H1); 44˚ C; productive haplomer duplex (H1 :: H2): 38˚ C.

Supplementary Tables

Supplementary Table S1. Peptide Modifications

Peptide Name	Peptide Function	Peptide Sequence	Modification	Modification structure	Source [b]	Purity Level ≥%	Molecular Weight	Relevant Figure (s)
A444	Thioester peptide mimotope fragment for NCL[a]	Azide-SGGGQLGPYELWEL-[phenyl thioester]	N-terminal azide; C-terminal phenyl thioester	N-terminal azidoacetic acid; C-terminal (C=O)-S-Phe	Anaspec	96	1681.0	1, S3
A423	Cysteine peptide mimotope fragment for NCL[a]	CHGGGSK-(Azide)	(N-terminal cysteine); C-terminal azide	C-terminal azidolysine	Anaspec	96	669.7	1, S3
Biotin-Jmim	Full mimotope; SA-binding [c]	Biotin-SGGGSGGGQLGPYELWELSH	N-terminal biotin	Direct biotinyl N-terminal amide	BioSynthesis	90	2214.33	S2, S13
Az-Jmim	Original mimotope; click reactions	Azide-SGGGSGGGQLGPYELWELSH	N-terminal azide	N-terminal azidoacetic acid	BioSynthesis	95	1870.04	S3
Biotin-S11C-Jmim	Mimotope with S11C cysteine substitution; SA-binding [c]	Biotin-SGGGSGGGQLGPYELWELCH	N-terminal biotin	Direct biotinyl N-terminal amide	BioSynthesis	95	2230.4	S1, S13
Biotin-S11A-Jmim	Mimotope with S11A alanine substitution; SA-binding [c]	Biotin-SGGGSGGGQLGPYELWELAH	N-terminal biotin	N-terminal amide with biotinyl-6-aminohexanoic acid	Vivitide	95	2053.3	S13

Footnotes to Table S1.

[a] Native Chemical Ligation

[b] Peptides analyzed by manufacturers for purity levels by HPLC.

[c] Streptavidin-binding

Supplementary Table S2. Oligonucleotide Sequences and Modifications

Oligonucleotide Name	Oligonucleotide Sequence [a]	Oligonucleotide Backbone	Modification	Modification structure / linker	Source [b]	Purification	Molecular Weight	Relevant Figure(s)
439	GTCCAGATGTCTTTGC	DNA	3’-DBCO [b]	C4 linker	IDT	HPLC	5390.7	1.2
440	TTTCTTCAGGACACAGC	DNA	5’-DBCO [b]	C4 linker	IDT	HPLC	5640.9	1.2
441	GCTGTGTCCTGAAGAAA	DNA	3’-DBCO [b]	C4 linker	IDT	HPLC	5762.0	1.2
Temp35	AGCTGTGTCCTGAAGAAAGCAAAGACATCTGGACA	DNA	-	-	IDT	Desalt	10901.1	1
Pye-A	UUUCUUCAGGACACAG	2’-O-methyl RNA	5’-pyrene	Pyrene-C6-amino linker	TriLink	HPLC	5657.5	3
Pye-B	UCCAGAUGUCUUUGC	2’-O-methyl RNA	3’-pyrene	Pyrene-C6-amino linker	TriLink	HPLC	5291.2	3
Lk-HPV1	**ACTCGAGACGTCTC**CTTGTCTTTGCTTTTCTTCAGGACACAGTGGC**GAGACGTCTCGAGT**	DNA	5’ -hexynyl	Linear alkyne, C6 linker	IDT	Desalt	18575.0	4, 6
Lk-HPV2	TTTGACGTCTCGAGT	DNA	3’-azide	3’-amino-NHS azide [b] conjugate	IDT	HPLC	4924.3	4, 6
Lk-EBV1	TTCG**ACTCGAGACGTCTC**CTTCCTGCCCCTCCTCCTGCTCC**GAGACGTCTCGAGT**	DNA	5’ -hexynyl	Linear alkyne, C6 linker	IDT	Desalt	16812.9	5, S11
Lin-HPV1	TCAGGACACAGTGGC	DNA	5’ -hexynyl	Linear alkyne, C6 linker	IDT	Desalt	4762.1	5, S10
Lin-HPV2	TGTCTTTGCTTTTCT	DNA	3’-azide	3’-amino-NHS azide [c] conjugate	IDT	HPLC	4856.3	5, S10

Supplementary Table S2. Oligonucleotide Sequences and Modifications, Continued

Oligonucleotide Name	Oligonucleotide Sequence [a]	Oligonucleotide Backbone	Modification	Modification structure / linker	Source [b]	Purification	Molecular Weight	Relevant Figure(s)
Lk2	GACGTCTCGAGTTCTT	DNA	3'-azide	3'-amino-NHS azide [c] conjugate	IDT	HPLC	5213.5	5, S11
EBN-Temp1	AGTTGCAGGAGCAGGAGGAGG GGCAGGAGCAGGAG	DNA	-	-	IDT	Desalt	11169.2	5
HPV-Rtemp1	AAGCCACUGUGUCCUGAAGAA AAGCAAAGACAUC	RNA	-	-	IDT	Desalt	10940	6
RTemp32	CUGUGUCCUGAAGAAAGCAAA GACAUCUGGAC	RNA	-	-	IDT	Desalt	10290	S5
439M	GUCCAGAUGUCUUUGC	2'-O-methyl RNA	3'-DBCO [b]	C4 linker	IDT	HPLC	5787.0	S5
440M	UUUCUUCAGGACACAGC	2'-O-methyl RNA	5'-DBCO [b]	C4 linker	IDT	HPLC	6081.2	S5
AK1	pCTTGTCCAGC^{Me}	DNA	3'-alkyne	5'-phosphate / 3'-(5-methyl dC) / 3'-propargyl	UWBC [d]	HPLC	3144.0	S6
AZ1	TGGACCATCT	DNA	5'-azide	5'-iodo dT conversion to azide	TriLink	HPLC	3106.0	S6
RTemp30	GAAAUAGAUGGUCCAGCUGGA CAAGCAGAA	RNA	-	-	IDT	Desalt	9420.0	S6
Lk-HPVscr1	GACAGCACCTTCGTCTTGTCTT TGCTTTTCTTCAGGACACAGTG GCGGGCTGCGACAATT	DNA	5' -hexynyl	Linear alkyne, C6 linker	IDT	Desalt	18575.0	S7

Supplementary Table S2. Oligonucleotide Sequences and Modifications, Continued

Oligonucleotide Name	Oligonucleotide Sequence [a]	Oligonucleotide Backbone	Modification	Modification structure / linker	Source [b]	Purification	Molecular Weight	Relevant Figure(s)
Lk-HPVscr2	ACTCGAGACGTCTCCTTGTCTT TGCTTTTCTTCAGGACACAGTG GCGGGCTGCGACAATT	DNA	5' -hexynyl	Linear alkyne, C6 linker	IDT	Desalt	18575.0	S8
Lk2A	CCATACTCTTCCATACGACGTC TCGAGTTCTT	DNA	3'-azide	3'-amino-NHS azide [c] conjugate	IDT	HPLC	10011.6	S11
Lk2B	CCATACTCTTCCATACGACGTC TCGAGTCGAA	DNA	3'-azide	3'-amino-NHS azide [c] conjugate	IDT	HPLC	10054.7	S11
Lk-1068M	**ACUCGAGACGUCUC**CUUGUC UUUGCUUUUCUUCAGGACACA GUGGC**GAGACGUCUCGAGU**	2'-O-methyl RNA	5'-pyrene	Pyrene-C6-amino linker	TriLink	HPLC	20399.2	7, S12
Lk-1069M	UUUGACGUCUCGAGU	2'-O-methyl RNA	3'-pyrene	Pyrene-C6-amino linker	TriLink	HPLC	5390.4	7, S12
Temp30	GCCACTGTGTCCTGAAGAAAA GCAAAGACA	DNA	-	-	IDT	Desalt	9242.1	7
Lk-HPV21Db	**GCCACTGTG**CTTTGTCTTTGCT TTTCTTCAGGA**CACAGTGGC**	DNA	5'-DBCO [b]	C4 linker	IDT	HPLC	13339.9	8. 9
Lk-s9Db	CACAGTGGC	DNA	3'-DBCO [b]	C4 linker	IDT	HPLC	3251.4	8, 9
HPV-temp56	TAACTGTCAAAAGCCACTGTGT CCTGAAGAAAAGCAAAGACATC TGGACAAAAAGC)	DNA	5'-biotin	5'-amide	IDT	Desalt	17363.2	8
Lk-1120M	**ACUCGAGACGUC**CACUUUCUU CAGGACACAGUGGCCAC**GACG UCUCGAGU**	2'-O-methyl RNA	5'-pyrene	Pyrene-C6-amino linker	TriLink	HPLC	17103.3	S12

Supplementary Table S2. Oligonucleotide Sequences and Modifications, Continued

Oligonucleotide Name	Oligonucleotide Sequence [a]	Oligonucleotide Backbone	Modification	Modification structure / linker	Source [b]	Purification	Molecular Weight	Relevant Figure(s)
2F-HPVwt	CTTACCTAATGCTGTTTATATCA TCGTTCTGT	DNA	5', 3' 6-FAM	5', 3'-amide	IDT	Desalt	10534.1	9
Temp35.2	GCTGTGTCCTGAAGAAAAGCA AAGACATCTGGACA	DNA	-	-	IDT	Desalt	10901.1	8, S12
RTemp35.2	GCUGUGUCCUGAAGAAAAGCA AAGACAUCUGGACA	RNA	-	-	IDT	Desalt	11300.0	S12

Footnotes to Table S2.

[a] Stem-forming sequences for Lk-NATS haplomers shown in bold

[b] Dibenzocyclootyne

[c] NHS = N-hydroxysuccinimide

[d] University of Wisconsin-Madison Biotechnology Center

Supplementary Table S3. Lk-NATS Structural Variables

1st haplomer name (length)	NA Backbone	1st haplomer Modifications	2nd haplomer name (length)	2nd haplomer Modifications	Stem length	Loop length	1st haplomer spacer / hinge(s) lengths [a]	2nd haplomer hybridization duplex length	Target length [b]	Relevant Figure(s)
Lk-HPV1 (60)	DNA	5'-hexynyl	Lk-HPV2 (15)	3'-azide	14	30	2 (5' stem: loop)	12	30	4, S7, S8
Lk-EBV1 (55)	DNA	5'-hexynyl	Lk2 (16)	3'-azide	14	21	4 (5') / 2 (5' stem: loop)	12	21	5, S9, S11
Lk-EBV1 (55)	DNA	5'-hexynyl	Lk2A (33)	3'-azide	14	21	(" ")	12	21	S11
Lk-EBV1 (55)	DNA	5'-hexynyl	Lk2B (33)	3'-azide	14	21	(" ")	16	21	S11
Lk-HPV21Db (42)	DNA	5'-DBCO	Lks9Db (9)	3'-DBCO	9	21	3 (5' stem: loop)	9	30 [shared stem]	8, 9
Lk-1068M	2'-O-methyl RNA (60)	5'-pyrene	Lk-1069M (15)	3'-pyrene	14	30	2 (5' stem: loop)	12	30	7, S12
Lk-1120M	2'-O-methyl RNA (50)	5'-pyrene	Lk-1069M (15)	3'-pyrene	12	20	3 (5' stem: loop) / 3 (3' loop: stem)	12	20	S12

Footnotes to Table S3.

Excludes control test oligonucleotides with scrambled stem regions.

Each row of the Table shows a specific First haplomer with a companion Second haplomer combination.

[a] Spacers / hinges indicate additional short sequences added at junctions between functional segments. 5' stem : loop indicates the hinge / spacer insertion at the junction between the 5' stem and the beginning of the loop sequence; 3' loop : stem indicates the hinge / spacer insertion at the junction between the 3' end of the loop and the beginning of the 3' stem sequence. The indicated total 1st haplomer lengths thus = (2 x [stem length] + loop + hinge / spacers).

[b] Target length is equivalent to the length of the loop region of the First Locked-NATS haplomer.

Supplementary Figures

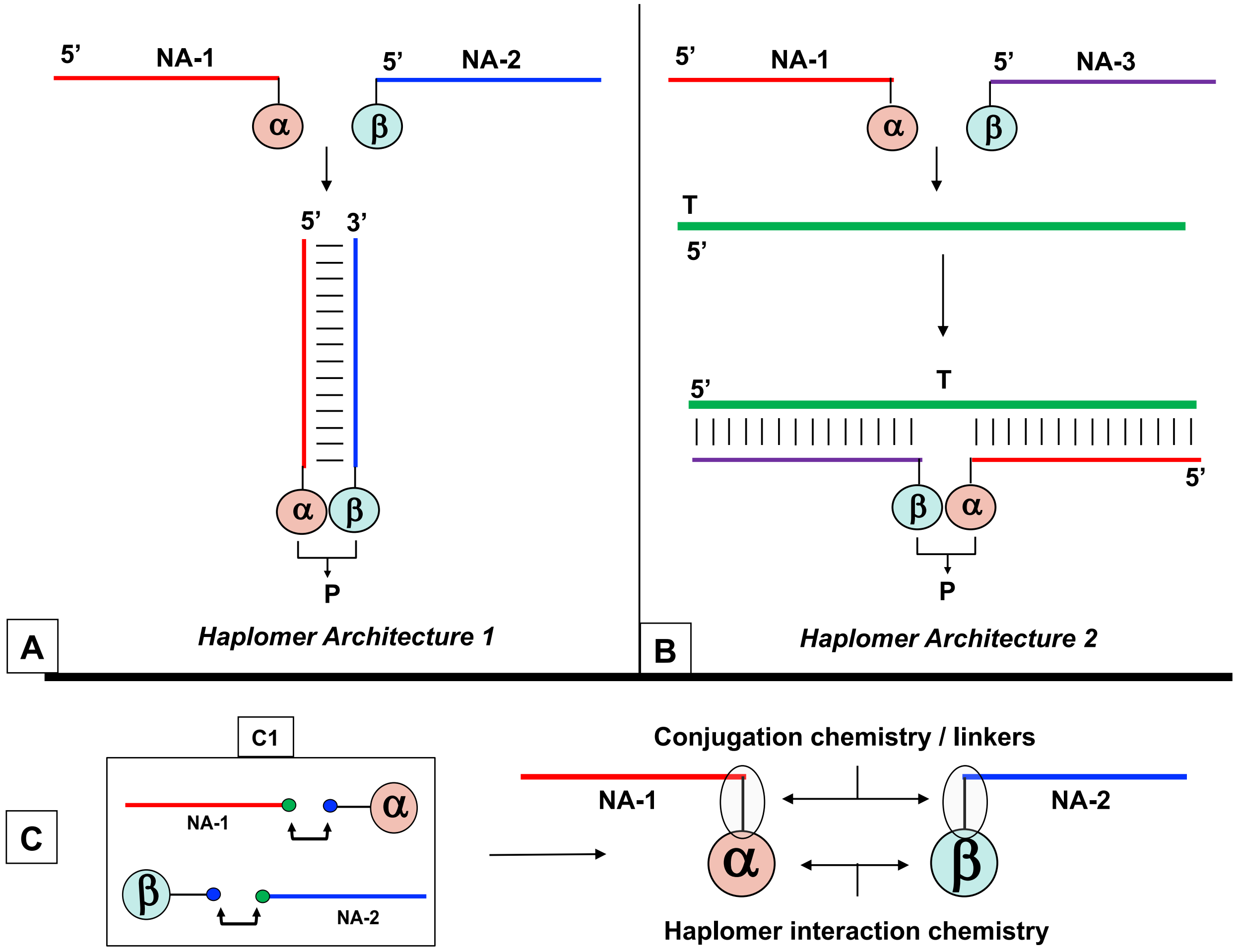

Supplementary Fig. S1. Structures of NATS component "haplomers" and relevant interactions. **A**, Two haplomers with complementary nucleic acid components NA-1 and NA-2, with mutually reactive moieties α and β conjugated to their 3' and 5' ends, respectively. **B**, Two haplomers mutually hybridizing in spatial proximity on a common template. Here the same α / β reactive moieties are used, but where the β moeity is conjugated with a different nucleic acid sequence (NA-3) complementary to the template. Both configurations promote spatial proximity between the α / β moeities and their resulting reaction towards product P. **C**, Schematic depiction of the requirement for two independent molecular reactions for successful NATS: the site-specific coupling between reactive α and β moieties and nucleic acids, and the designed reactivity between the α / β moieties themselves. Subpanel C1 depicts the initial separate coupling between reactive groups on both nucleic acid segments and α and β moieties, prior to templating of the nucleic acids that promotes molecular proximity and reaction acceleration between α and β.

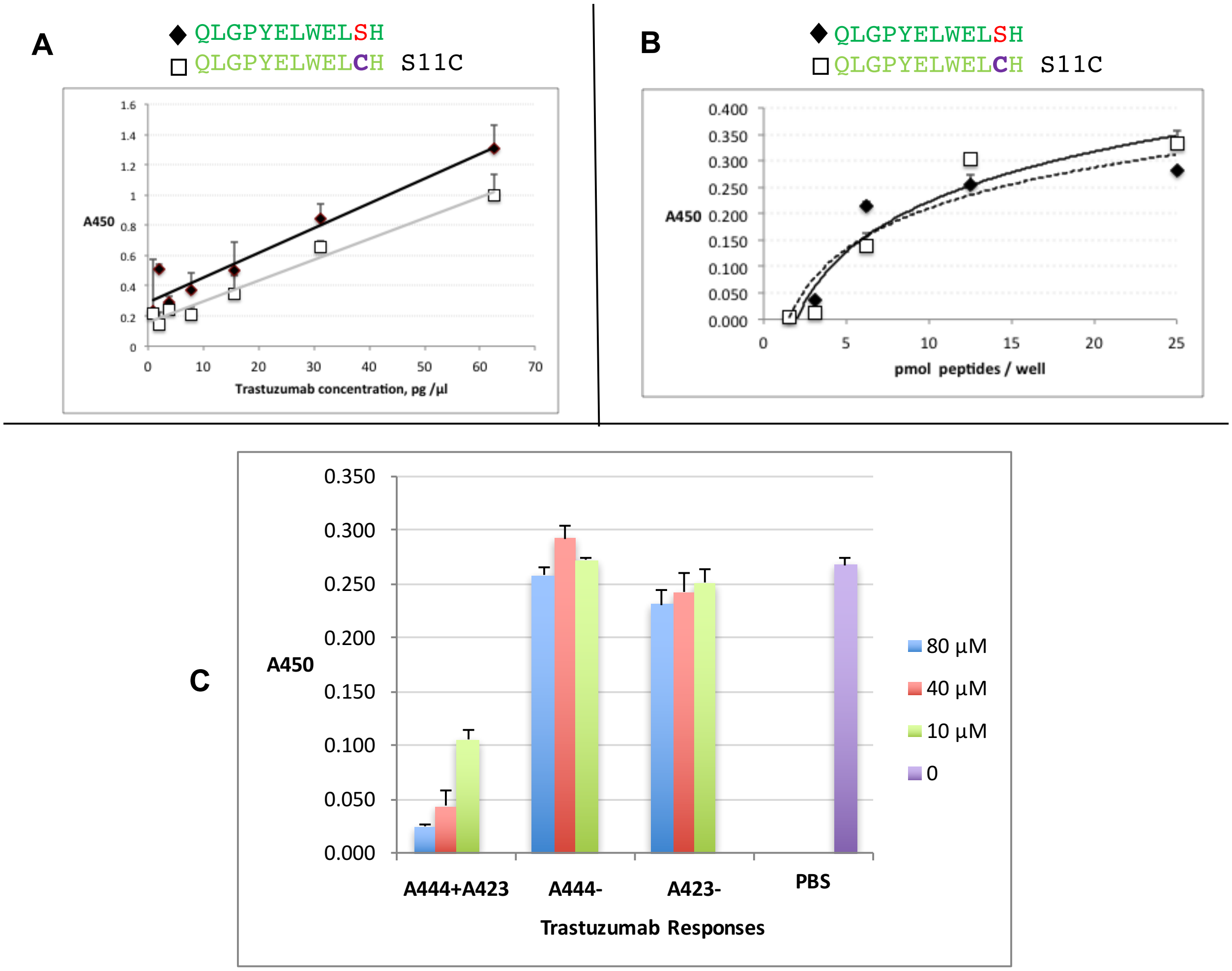

Supplementary Fig. S2. Reconstitution of a trastuzumab mimotope S11C variant by NCL. Reactivity of a trastuzumab (Herceptin) mimotope and its S11C variant, and reconstitution of the S11C mimotope by NCL. **A**, ELISA testing for binding of trastuzumab with original (Jmim) vs. S11C mimotopes, by saturating amounts of biotinylated mimotope peptides in each well of a streptavidin plate against titration of antibody levels. Absorbance read-out is at 450 nm (Methods). **B**, ELISA testing binding of trastuzumab with original vs. S11C mimotopes, with constant antibody (2 ng / µl) against titration of biotinylated peptide levels in streptavidin plates. **C**, Blocking of trastuzumab binding to NCL-assembled S11C mimotope product. Modified peptide segments of the S11C mimotope (Fig. 2C) were incubated either separately or together in phosphate-buffered saline (PBS, at 400 µM) with 1 mM TCEP for 48 hr / RT, and incubated in specific dilutions with a constant concentration of trastuzumab (2 ng / µl) for 2 hr RT. Each preparation and appropriate controls were tested for trastuzumab binding to intact biotinylated S11C peptide immobilized on ELISA plates by streptavidin.

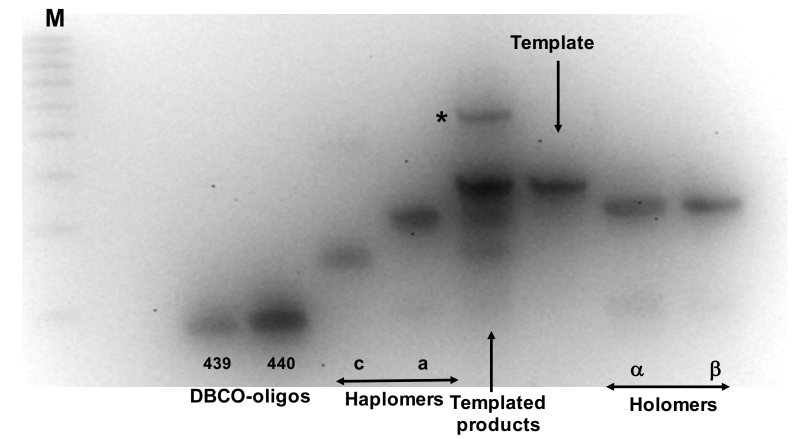

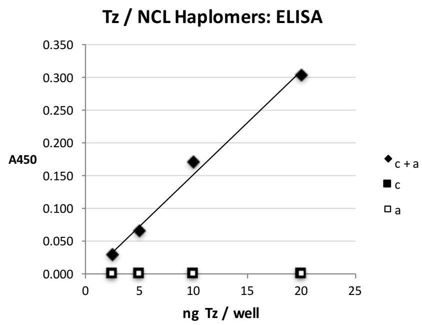

Table 1

Molecular Species / (Code)	Molecular weight (kD)	Sequence / structure *
Haplomer **c** (439-A423)	6028	5' GTCCAGATGTCTTTGC-<DBCO-Azide>-**KSGGGHC /** N-term.
Haplomer **a** (A444-440)	7321	3' CGACACAGGACTTCTTT-<DBCO-Azide>-**SGGGQLGPYELWEL**-phenyl thioester / C-term.
Template (#211)	10822	5' AGCTGTGTCCTGAAGAAAGCAAAGACATCTGGACA
Peptide A423	670	**CHGGGSK**(azide)-NH2 / C-term.
Peptide A444	1681	2-azidoacetic acid – **SGGGQLGPYELWEL**-phenyl thioester / C-term.
Oligo #439	5358	5' GTCCAGATGTCTTTGC-[C6]-DBCO
Oligo #440	5640	5' DBCO-[C6]-TTTCTTCAGGACACAGC

Table 2

Holomer Species	Molecular weight (kD)	Sequence / structure *
α-Holomer	7728	5' [oligo-439]-<DBCO-Azide>-**SGGGQLGPYELWELSH /** C-term.
β-Holomer	7510	5' [oligo-440]-<DBCO-Azide>-**SGGGQLGPYELWELSH /** C-term.

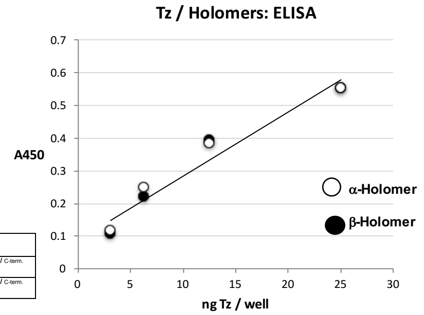

Supplementary Fig. S3. Haplomer and holomer configurations and templating.

Top left panel: 15% 8 M urea gel for samples of precursor DBCO-oligonucleotides #439 and #440, and their reaction products with activated peptide fragments to form haplomers [**c**] and [**a**] (Table 1) or whole mimotopes to form holomers α and β (Table 2). The haplomers **c** and **a** were subsequently incubated with a mutually complementary template (Fig. 1). Reactions were performed at room temperature for 16 hr, and treated with 10 mM TCEP for ≥2 hr 25˚ C. Samples of each reaction were denatured in formamide before gel analysis and staining with SYBR-Gold. The NCL product band was well-resolved from all other components and indicated by an asterisk. M = 100 / 10 size markers (IDT). The haplomers were also tested for *in situ* reactions on solid-phase templates in ELISA assays for trastuzumab (Tz) recognition. Haplomer preparations (20 pmol / µl) were treated for 16 hr with 10 mM TCEP before diluting to to 0.2 pmol / µl in 0.1 mM TCEP prior to assay. A 5'-biotinylated version of the 211-template (Table 1) was initially bound in excess (100 pmol / well) to wells of streptavidin plates (R&D Systems), the plates washed, and then treated with **c** and **a** separately or in combination in multiple wells, with each haplomer singly or combination added at a final of 2 pmol / well in 100 µl. After allowing 15 min for haplomer / template hybridizations, plates were washed, reconstituted with 100 µl PBS, and incubated 1.5 hr at room temperature for NCL reactions to occur. After washing, dilution series of trastuzumab (BioVision) were added for 1 hr (room temperature), before treatment with a secondary anti-human kappa chain-HRP

conjugate (Southern Biotech), and development with TMB HRP reagent (BioLegend). A representative ELISA result in shown in the panel Tz (trastuzumab) NCL Haplomers: ELISA, showing that no measurable signals are seen from either haplomer in isolation, but are readily detectable from the combination of Haplomer [**c]** and Haplomer [**a]**. Holomers were assayed in a similar manner as for the haplomers (panel Tz (trastuzumab) Holomers: ELISA), where the responses were almost identical for the same full mimotope peptide separately conjugated to the two oligonucleotides #439 and #440 (α-holomer and β-holomer respectively) where both are mutually complementary to the longer solid-phase template. Standard deviations for data points were <10 % of average values from replicate determinations. Further peptide and oligonucleotide details are provided in Supplementary Tables S1 and S2, respectively.

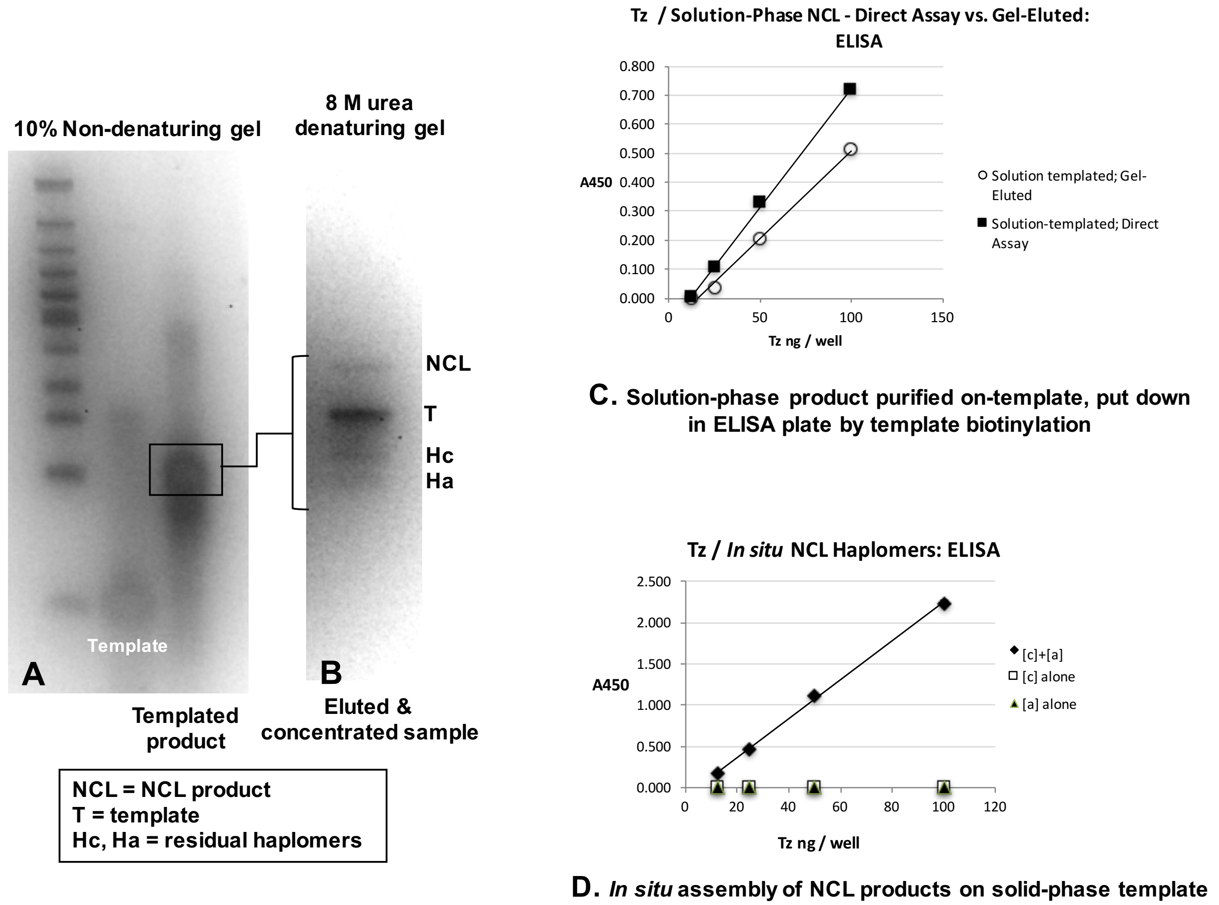

Supplementary Fig. S4. Solution-phase NCL templating and complex elution, and ELISA with biotinylated template. Haplomers [**c**] and [**a**] and a 5'-biotinylated version of template as in Fig. 1 were incubated in solution (10 pmol / µl, in equimolar ratios in x1 PBS pH 7.4) for 16 hr / 25˚ C, after which samples were tested on a 10% nondenaturing acrylamide gel. The template band hybridized to the haplomers showed a pronounced mobility shift relative to the template in isolation (Gel panel A). The template : haplomer complex was excised (black rectangle) and eluted from the gel as described (1), and tested on a 15% 8 M urea denaturing gel (Gel panel B), where the complex band was shown to be composed of the original template, excess haplomers, and a higher-molecular NCL product. Both the direct solution-phase products and the excised gel product complex were tested by ELISA for trastuzumab (Tz) functional recognition, by binding the biotinylated template strand and accompanying hybridized haplomers / haplomer reaction products to wells of a streptavidin plate (ELISA panel C). In parallel, a control *in situ* NCL reaction was performed in the ELISA plate in the same manner as for Fig. 2; Supplementary Fig. S3, with the same haplomer codes (ELISA panel D; (haplomer [a] = oligonucleotide #440 click conjugated to peptide A444; haplomer [b] = oligonucleotide #441 click conjugated to peptide A423; haplomer [c] = oligonucleotide #439 click conjugated to peptide A423; template = biotinylated

version of Temp35 5’-biotin- AGCTGTGTCCTGAAGAAAGCAAAGACATCTGGACA; where haplomers anneal and react on template in an Architecture 2 configuation [Supplementary Fig. S1]). Standard deviations for data points were <10 % of average values from replicate determinations.

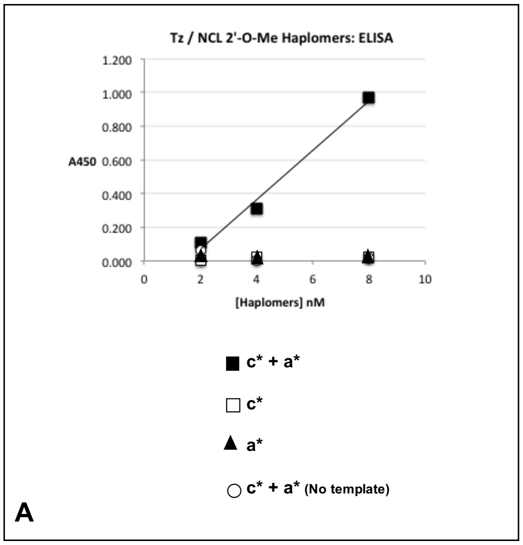

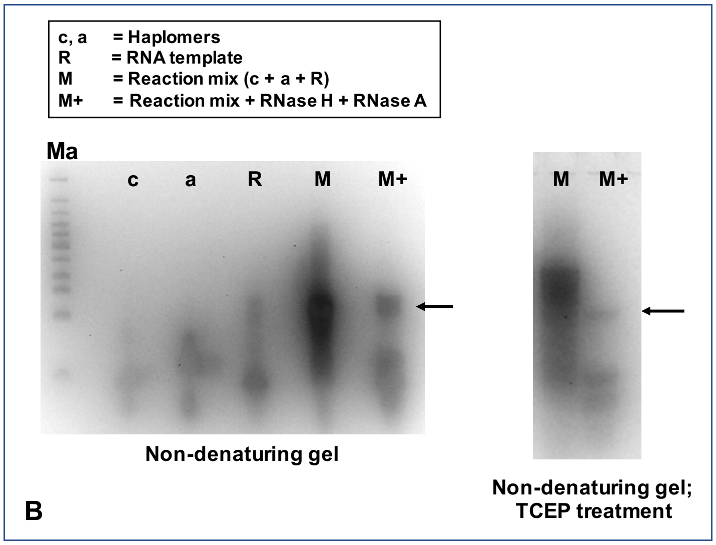

Supplementary Fig. S5. A, Generation of NCL product recognizable by trastuzumab (Tz) with 2'-O-methyl haplomers. NCL product was produced with the same structures as shown in Fig. 1A and 1B except for conversion of the nucleic acid components to 2'-O-methyl RNAs, while preserving the same sequences. Haplomer **c*** = equivalent structure as for Haplomer **c** (Fig. 1B): a 2'-O-methyl version of oligo-#439 (439M) conjugated with peptide A423 (Supplementary Tables S1, S2, Fig. 1C); **a*** = equivalent structure as for Haplomer **a** (Fig. 1A): 2'-O-methyl version of oligo-#440 (440M) conjugated with peptide A444 (Supplementary Tables S1, S2, Fig. 1C). ELISAs with conducted with or without a biotinylated template in streptavidin plate wells (as in Fig. 2) followed by hybridization with a haplomer dilution series, and after washing, treatment with a constant amount (2 µg / ml) of trastuzumab, and subsequent processing as usual (Methods). Standard deviations for data points were <15% of average values from replicate determinations. %. **B**, RNA templating for NCL product generation. Haplomers **c** and **a** (Fig. 1) were incubated in solution with a similar RNA version of template as used in Fig. 1C, Supplementary Fig. S2 (RTemp.32; CUGUGUCCUGAAGAAAGCAAAGACAUCUGGAC), all components equimolar with respect to each other (8.3 µM during the reaction) for 2 hr / 25˚ C in the presence of murine RNase inhibitor (New England Biolabs). Following this, the full reaction mixture was treated with excess RNase A and RNase H to digest both free RNA and RNA in RNA: DNA hybrids. Samples were then tested on 10% non-denaturing acrylamide gel, and samples of the full reaction mix were also run on a 12% non-denaturing gel following treatment with 50 mM TCEP (30 min). Horizontal arrows show the positions of the NCL products present only in the complete reaction mix. Further oligonucleotide details for 439M and 440M are provided in Supplementary Table S2.

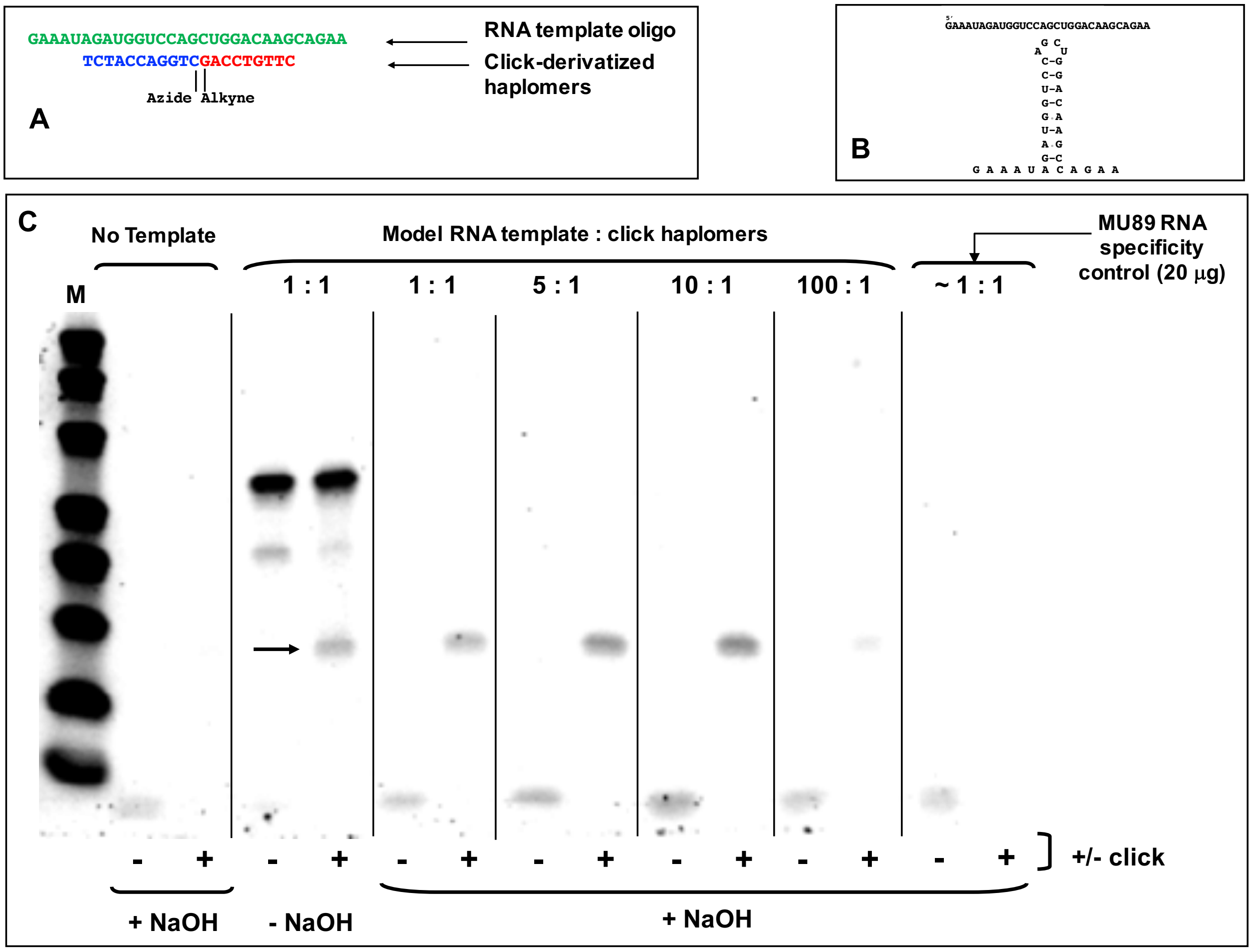

Supplementary Fig. S6. Haplomer product formation with an RNA template forming secondary structures; effects of template excess. DNA oligonucleotides equipped at their 5' or 3' ends with mutually reactive click groups (5' azide [AZ1] and 3' linear alkyne [AK1]; Panel A), were used with an RNA template where the click reaction was catalysed by univalent Cu(I), followed by denaturing gel electrophoresis and visualization by SYBR-Gold staining (Methods). The model template in this situation was an RNA oligonucleotide with predicted significant secondary structure (example in Panel B). To remove the background of RNA staining (especially when added at high molar excess), sodium hydroxide treatment was used in all lane sets (Panel C) except the first (1:1) molar ratio (+/- click pair). Arrow indicates the click product; M = 60/10 oligonucleotide molecular size markers (IDT). RNA from the MU89 melanoma cell line was used as a specificity control, where the calculated RNA : haplomer ratio was based on assuming average size of mammalian mRNA = 1500 bases. Further oligonucleotide details for AZ1 and AK1 are provided in Supplementary Table S2.

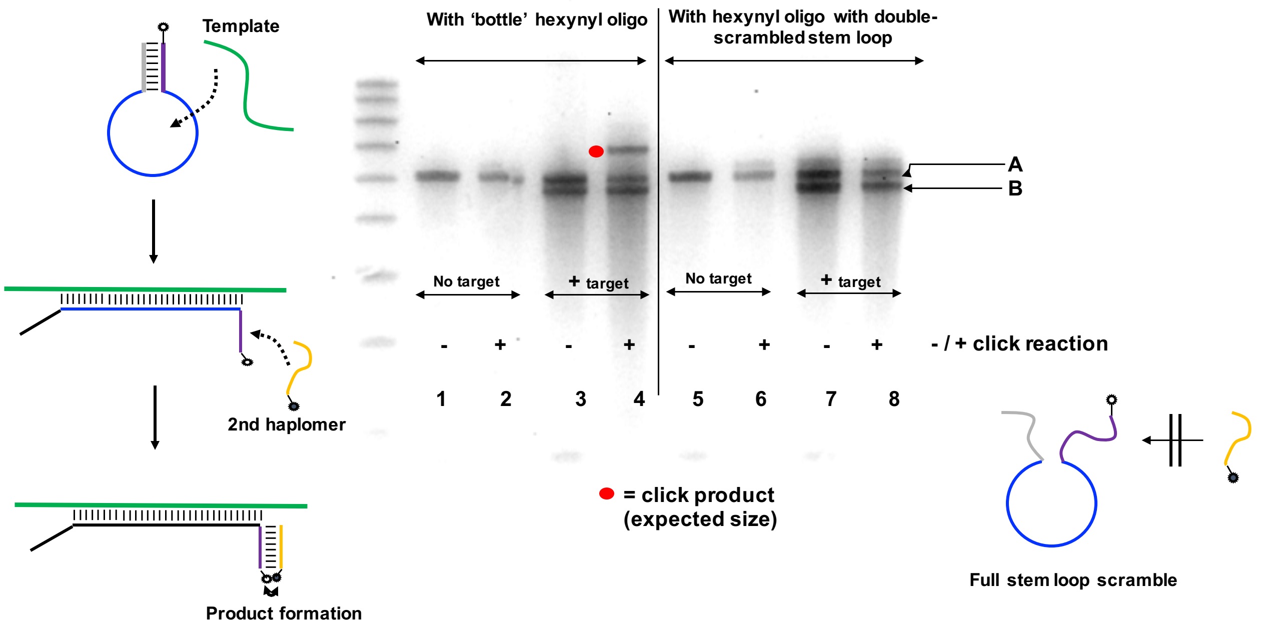

Supplementary Fig. S7. Locked-NATS Model Oligonucleotide Tests: Effect of scrambled stem loop. The specificity of the click product formation was probed by the use of an oligonucleotide version of the HPV sequence-targeting First haplomer bottle (Lk-HPVscr1) where both stem sequences were randomly scrambled, in comparison to the original bottle oligonucleotide (Fig. 6A). Experimental conditions were as above, except a single combined 30 minute incubation (with no pre-incubation) was used for all components. Following this incubation period, samples were subjected to +/- treatment for Cu(I) click reactions, as described (Methods). Samples were subsequently analyzed on a 10% denaturing gel with 8 M urea and stained with SYBR-Gold (Thermofisher). **A** band = Unreacted First haplomer bottle; **B** band = template band corresponding to HPV sequence. Results confirmed that for the original First haplomer bottle, click product was only seen in the presence of template (lanes 1 vs. 2; 3 vs 4; click product marked with a dot). No product was seen where both stem loops had the same base composition as for the original, but scrambled sequences (Lanes 5-8), demonstrating the requirement for specific templating in the reaction mediated by the click groups. Further oligonucleotide details for Lk-HPVscr1 are provided in Supplementary Table S2.

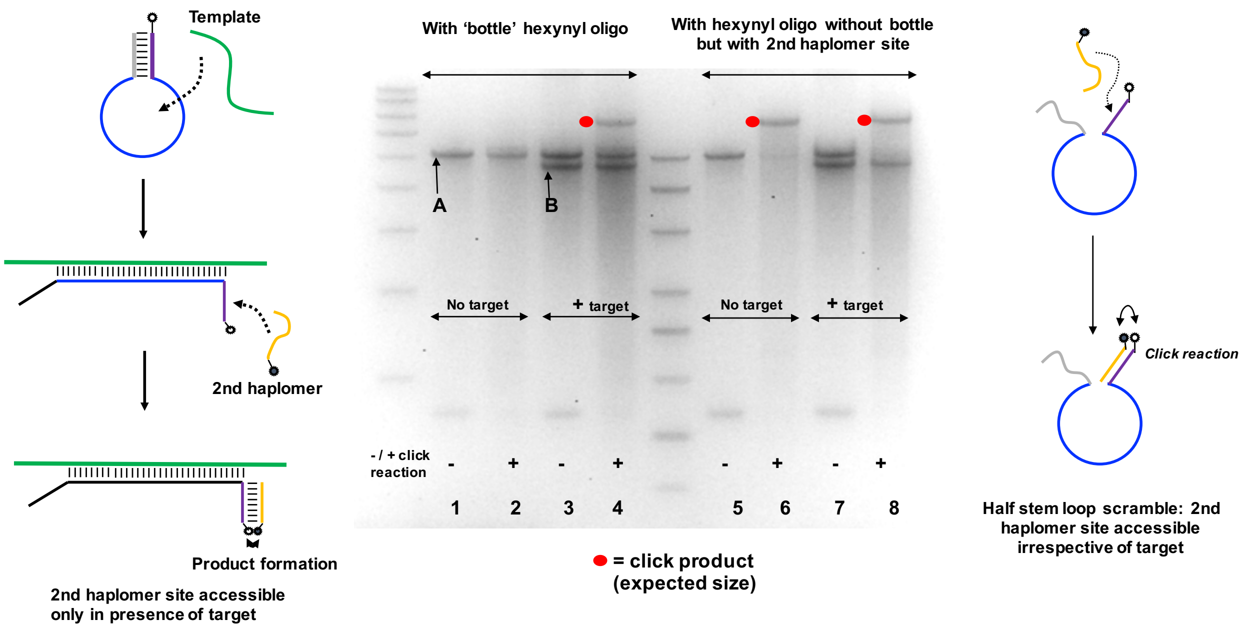

Supplementary Fig. S8. Locked-NATS Model Oligonucleotide Tests: Absence of bottle but presence of second haplomer site. In parallel with Supplementary Fig. S5, a further test of the locked NATS system used a 5' hexynyl First bottle haplomer where only one of the two complementary sequences forming the stem loop was scrambled (Lk-HPVscr2). In this case, the sequence complementary to the second haplomer was maintained, although no loop bottle could form. Both the original HPV first haplomer loop bottle Fig. 6A) and single-scrambled control were subjected to a 2 hr pre-incubation with or without a two-fold excess of HPV template, followed by an additional 1 hr incubation with the Second (azide) haplomer, and then standard +/- click reactions as described (Methods). Processed samples were tested on 15% 8 M urea gels. **A** band = Unreacted First haplomer bottle; **B** band = template band corresponding to HPV sequence. Click products are marked with dots in Lanes 4, 6, and 8. Further oligonucleotide details for Lk-HPVscr2 are provided in Supplementary Table S2.

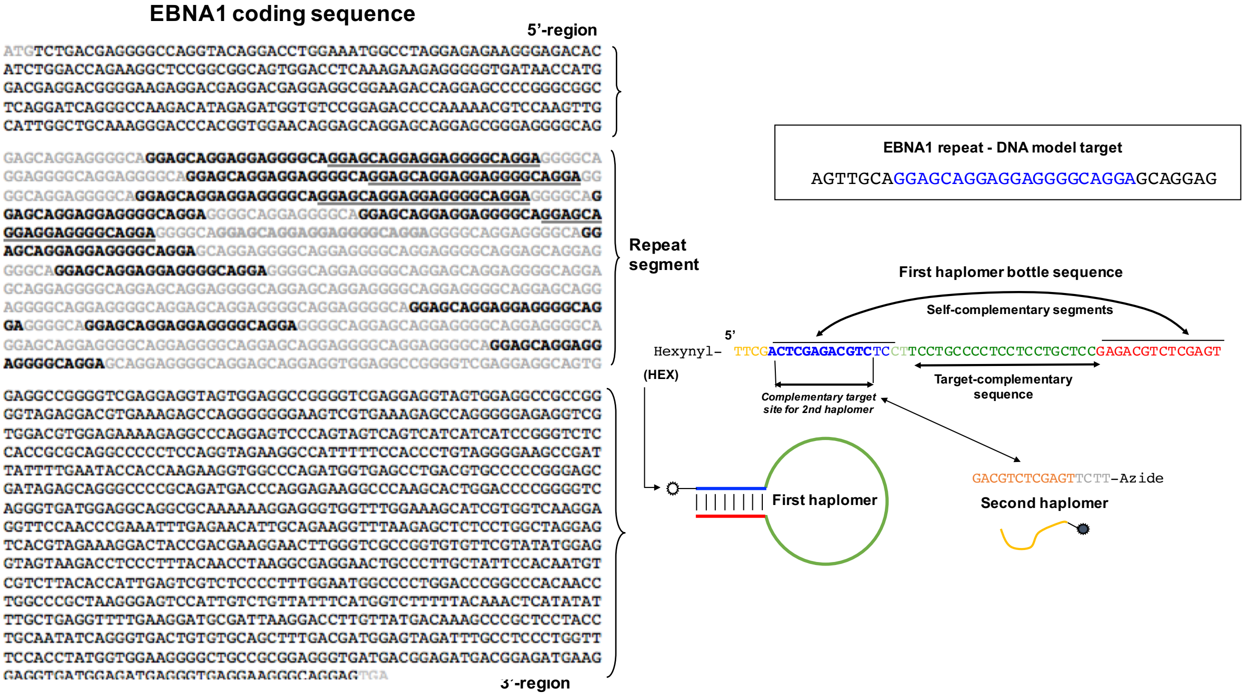

COMPOSITE

Supplementary Fig. S9. EBNA-1 coding sequence repeat motif, and Locked-NATS design. The coding sequence is shown divided into the N- and C-terminal segments separated by an extensive repetitive domain, where the targeted repeat motif is shown in bold (non-overlapping copies also underlined). The design of the Locked-NATS First and Second haplomers (Lk-EBV1 and Lk2 respectively) is in parallel with the HPV-haplomer for Fig. 4A, but with the loop region replaced with a complementary sequence of the EBNA-1 repeat region. Further details for Lk-EBV1 and Lk2 are provided in Supplementary Tables S2 and S3.

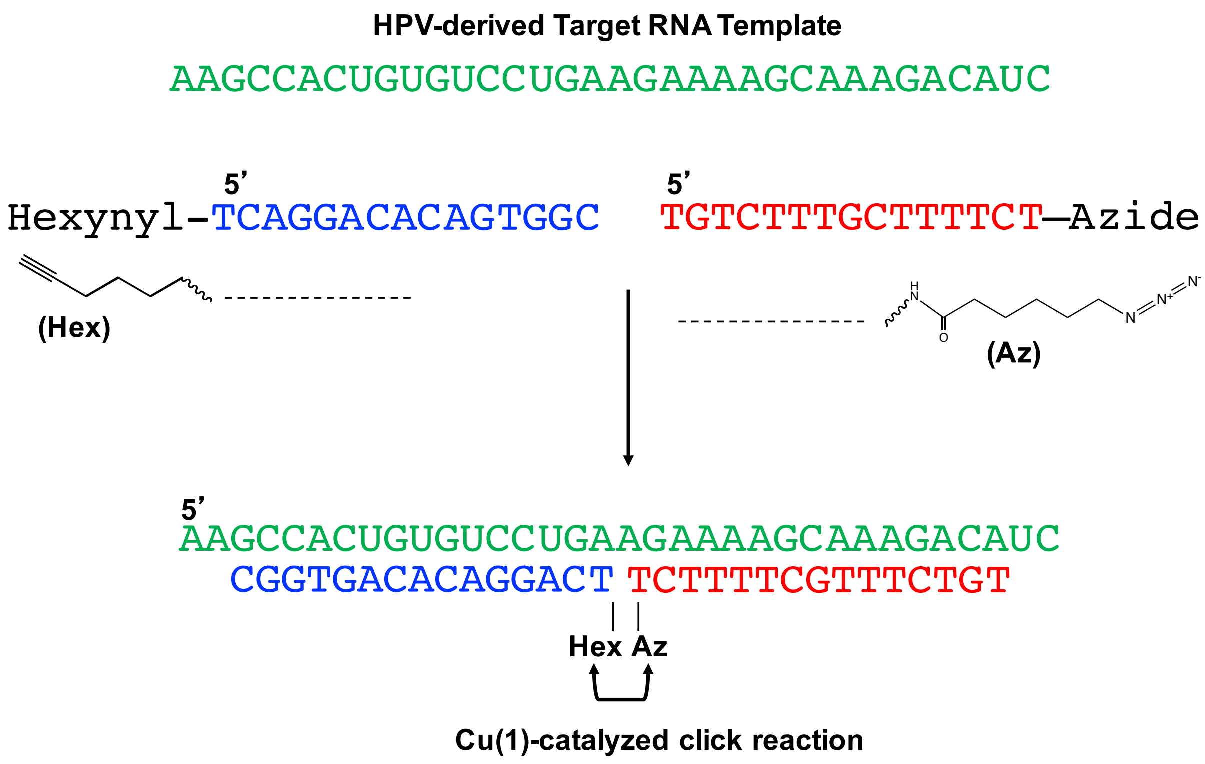

Supplementary Fig. S10. Linear click haplomers for HPV-derived RNA target sequence, with 5’-hexanyl (Linear-HPV1) or 3’-azide (Linear-HPV2). Structures are shown for the modified linear haplomers, and their configurations when annealed to the target sequence.

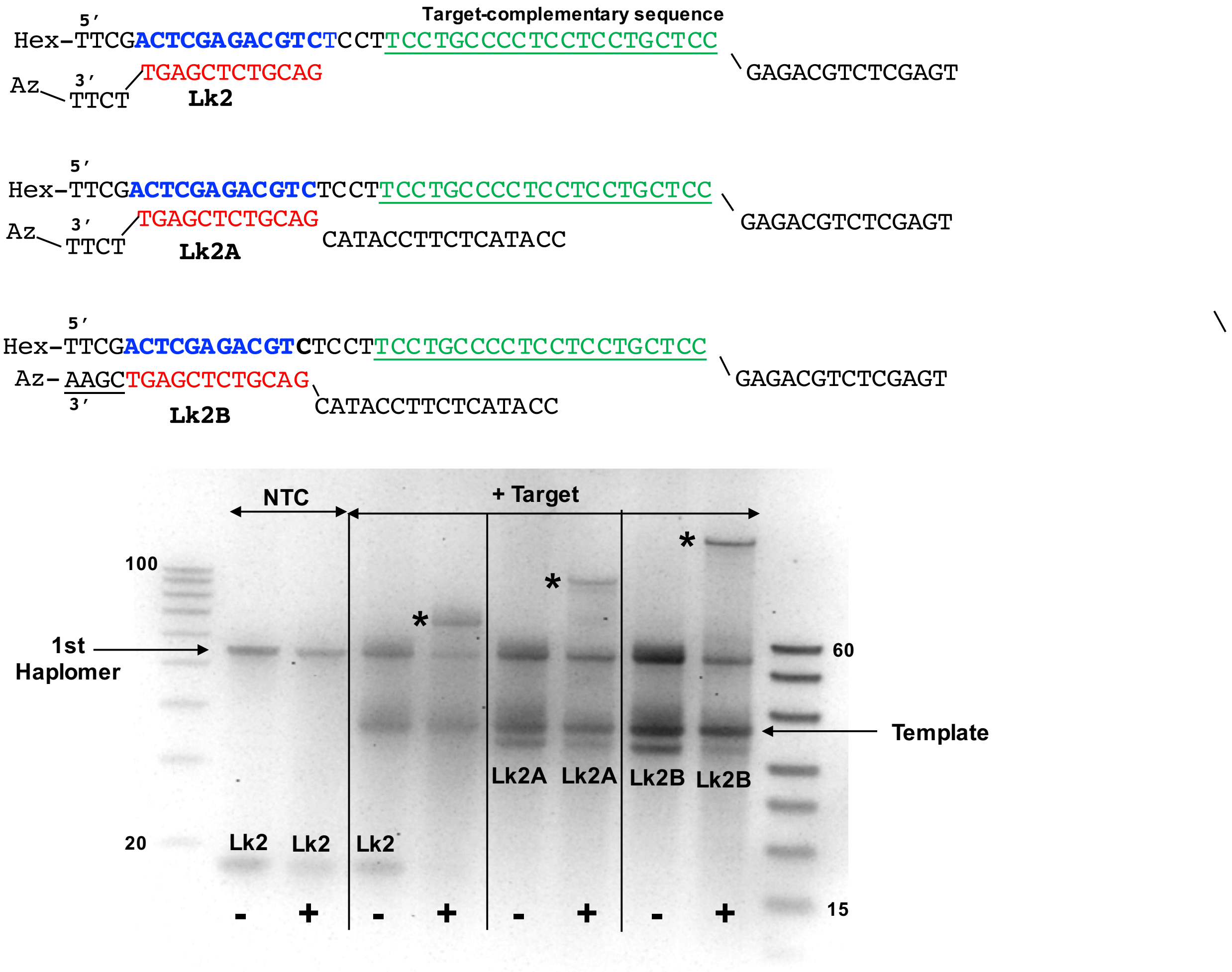

Supplementary Fig. S11. Locked-NATS with extended Second haplomers bearing 3’ azide (Az) modifications. The sequences show the First haplomer (with 5’ hexanyl, HEX) in an open state as occurs after annealing with a target sequence (in this case, the EBNA-1 EBV target as in Fig. 7, Supplementary Fig. S7). In each case, in the open state the First haplomer can anneal with a complementary Second haplomer (Lk2, Lk2A, or Lk2B). The Second haplomers Lk2 and Lk2A share the same 12-bp complementary region to the First haplomer, and a 4-base non-complementary segment at their 3’ ends. Both Lk2A and Lk2B share an extended 5’ end relative to Lk2, but the complementary region for Lk2B is extended to 15 bp to the 3’ end of the First haplomer. After incubations with or without target template, preparations were subjected to (-/+) click reactions (Materials and Methods) and tested on a 15% denaturing 8 M urea gel. Positions of the First (1st) haplomer, the Second haplomers (Lk2, Lk2A, and Lk2B), and target template are as indicated. NTC = No-target control. Positions of the Locked-NATS products arising from click reactions are shown with asterisks. Although the Second haplomers Lk2A and Lk2B were of the same size and gel mobilities, the product formed with the Lk2B showed aberrant

mobility, presumed to be due the formation of a denaturation-resistant stem-loop structure after hexanyl-azide click reaction. Further oligonucleotide details are provided in Supplementary Table S2 and S3.

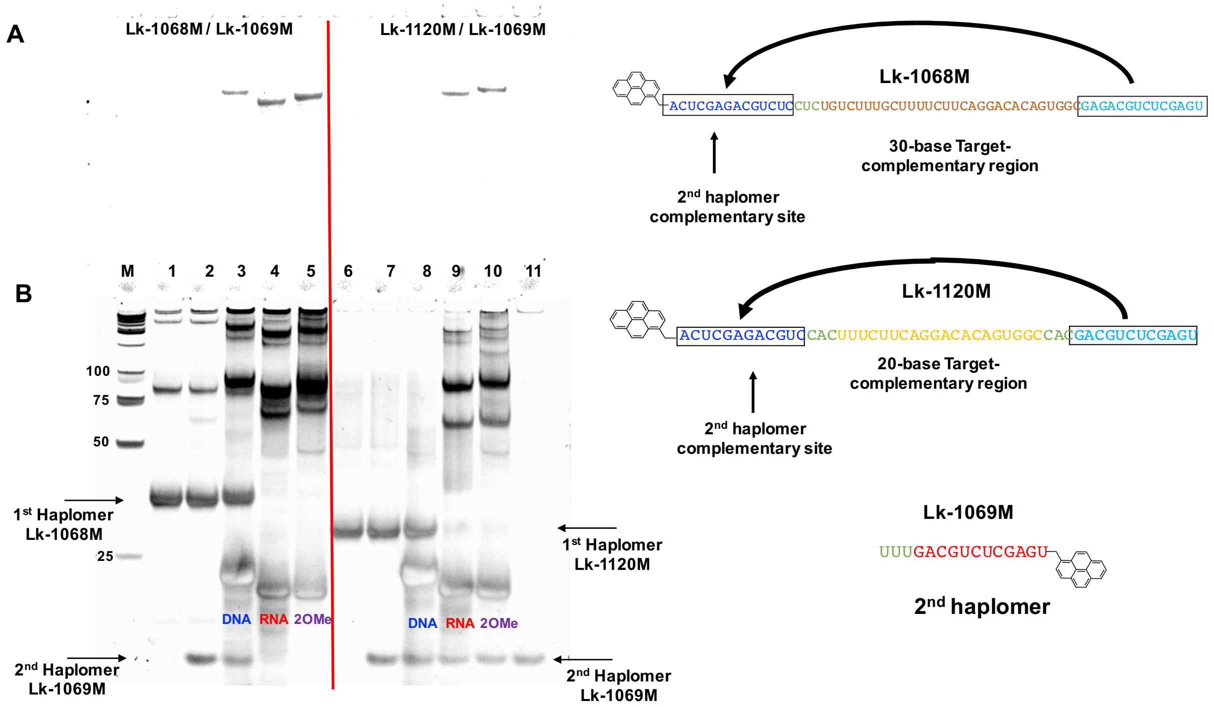

Supplementary Fig. S12. Pyrene haplomer activity and fluorescence in a non-denaturing 12% Tris-Glycine gel, with three templates of corresponding sequence but different backbones. **A** (Top panel) gel visualized under direct UV fluorescence (302 nm excitation), **B** (bottom panel) gel with the same samples (run on separate gel with identical conditions) stained with SYBR-Gold. Lane M, low-molecular weight markers (New England Biolabs) with sizes (bp) of the first 4 bands shown; Lanes 1-5: First pyrene haplomer Lk-1068M / Second haplomer Lk-1069M; Lanes 6-10: First pyrene haplomer Lk-1120M; Second pyrene haplomer Lk-1069M. Lanes 1, 6: First haplomers only; Lanes 2, 7: First and second haplomers without template. Lanes 3, 8: 35-meDNA template (GCTGTGTCCTGAAGAAAAGCAAAGACATCTGGACA), Lanes 4, 9: RNA template (GCUGUGUCCUGAAGAAAAGCAAAGACAUCUGGACA), Lanes 5, 10: 2'-O-methyl RNA template (2OMe; as for RNA except 2'-O-methyl backbone); Lane 11: Second haplomer Lk-1069M only. Further oligonucleotide details for the pyrene haplomers are provided in Supplementary Table S2; with their structures depicted on the right. (Open boxed sequences show segments forming stem-loops, gray-shaded boxes show regions complementary to Second haplomer Lk-1069M. Note the stem-forming and Lk-1069M complementary regions co-incide for First haplomer Lk-1120M).

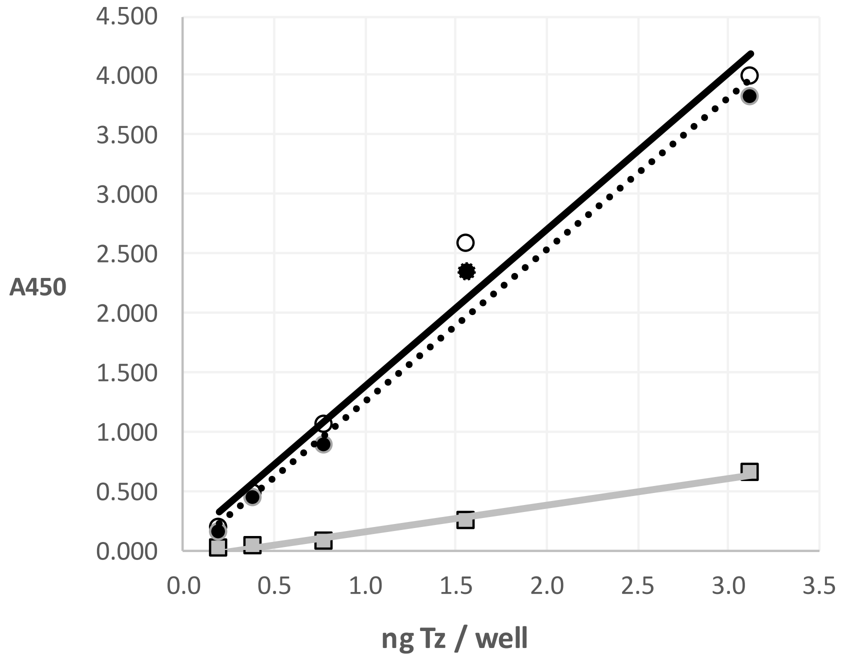

Supplementary Fig. S13. Comparison of original mimotope (Biotin-Jmim) with Biotin-S11C (used for NCL) and Biotin-S11A analogs by ELISA with trastuzumab (Tz) antibody. Conditions were as for Fig. 2A with saturating amounts of biotinylated peptides / well. Open circles, original mimotope (Jmim), filled circles, peptide S11C analogue; squares, peptide S11A analogue. Standard deviations for data points were ≤10 % of average values from replicate determinations. Further peptide details are provided in Supplementary Table S1.

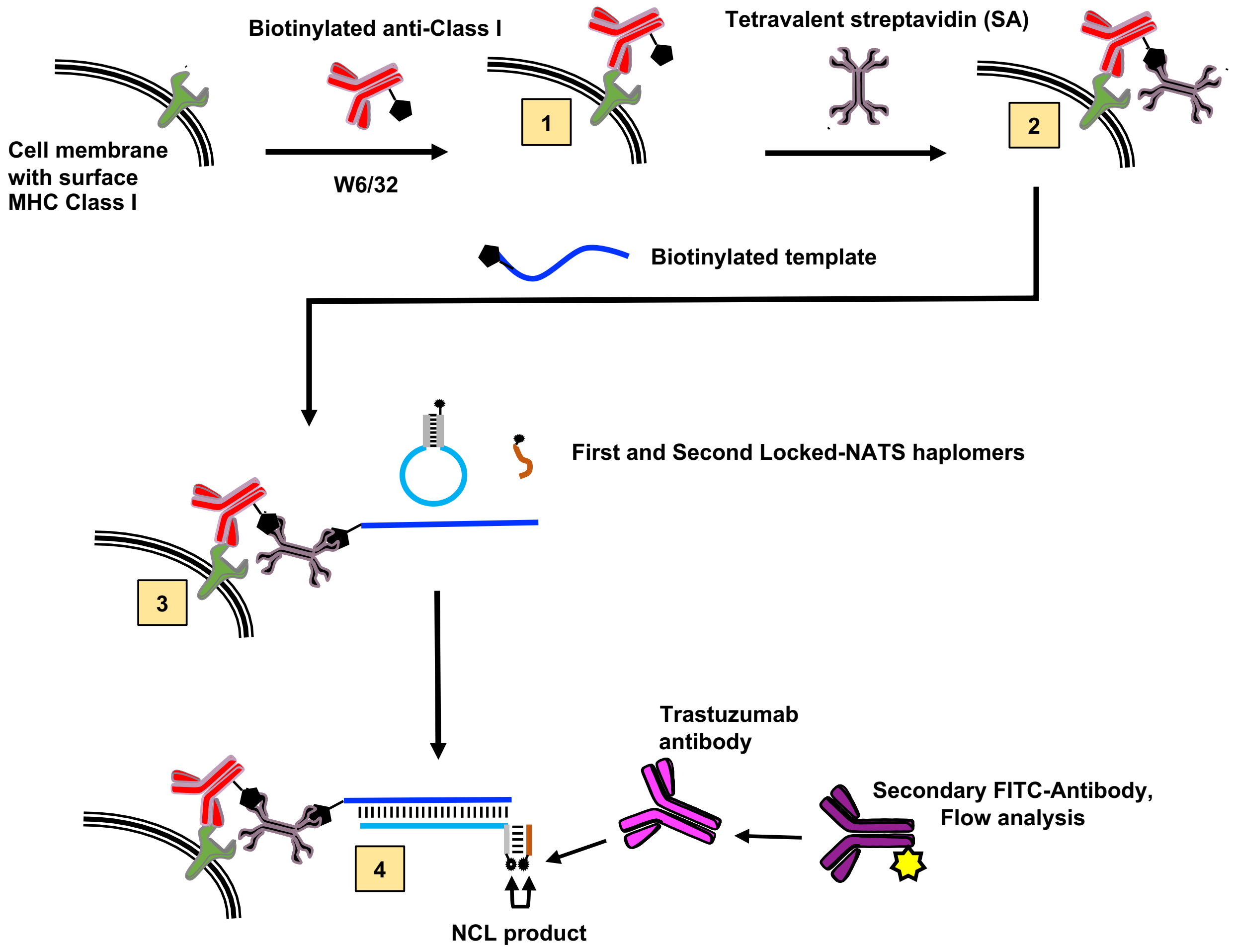

Supplementary Fig. S14. Schematic depiction of placement of cell surface template via anti-Class I MHC antibody and streptavidin, followed by haplomer hybridizations for NCL reactions. Steps: [1] Target cells are treated with biotinylated anti-Class I MHC antibody (W6/32, Thermo); [2] Cells are washed and treated with tetravalent streptavidin; [3] The streptavidin-bearing cells are then treated with biotinylated template, washed, and then given Lk-NATS First and Second haplomers derivatized with Native Chemical Ligation (NCL) thioester and cysteinyl mimotope fragments; [4] Final read-out is staining with trastuzumab, followed by secondary FITC-labelled anti-human kappa light chain antibody.

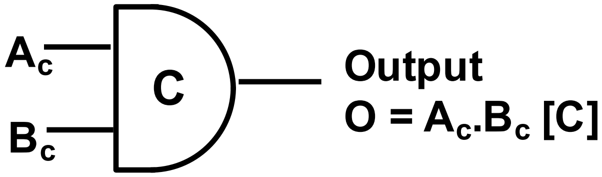

Inputs		Outputs
A_c	B_c	O
0	0	0
1	0	0
0	1	0
1	1	1

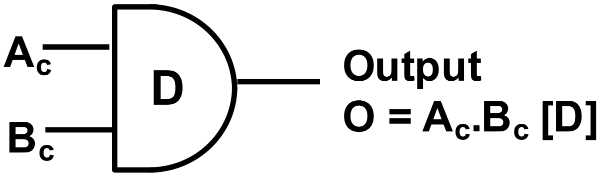

Inputs		Outputs
A_D	B_D	O
0	0	0
1	0	0
0	1	0
1	1	0

Supplementary Fig. S15. NATS as a conditional AND logic gate. Ac and Bc = a pair of haplomers specifically targeting a template C. As represented in the Truth Tables, if a binary haplomer pair is received simultaneously at a C gate, functional output is produced, whereas no output is elicited from a non-C gate, represented here by a D gate.

Reference

1. Jain, N., Blauch, L.R., Szymanski, M.R., Das, R., Tang, S.K.Y., Yin, Y.W. and Fire, A.Z. (2020) Transcription polymerase-catalyzed emergence of novel RNA replicons. *Science*, **368**.
